# Cross-border care and healthcare quality improvement in Europe: the MARQuIS research project

**DOI:** 10.1136/qshc.2008.029678

**Published:** 2009-01-26

**Authors:** R Suñol, P Garel, A Jacquerye

**Affiliations:** 1 Avedis Donabedian Institute, Autonomous University of Barcelona, and CIBER Epidemiology and Public Health (CIBERESP), Spain; 2 European Hospital and Healthcare Federation (HOPE), Brussels, Belgium; 3 Patient Safety and Risk Management in Health Care Systems, Department of Health Economy, School of Public Health, University of Brussels, Brussels, Belgium

## Abstract

Citizens are increasingly crossing borders within the European Union (EU). Europeans have always been free to travel to receive care abroad, but if they wished to benefit from their statutory social protection scheme, they were subject to their local or national legislation on social protection. This changed in 1991 with the European Court of Justice defining healthcare as a service, starting a debate on the right balance between different principles in European treaties: movement of persons, goods and services, versus the responsibility of member states to organise their healthcare systems. Simultaneously, cross-border cooperation has developed between member states.

In this context, patient mobility has become a relevant issue on the EU’s agenda. The EU funded a number of Scientific Support to Policies (SSP) activities within the Sixth Framework Programme, to provide the evidence needed by EU policy makers to deal with issues that European citizens face due to enhanced mobility in Europe. One SSP project “Methods of Assessing Response to Quality Improvement Strategies” (MARQuIS), focused on cross-border care. It aimed to assess the value of different quality strategies, and to provide information needed when: (1) countries contract care for patients moving across borders; and (2) individual hospitals review the design of their quality strategies. This article describes the European context related to healthcare, and its implications for cross-border healthcare in Europe. The background information demonstrates a need for further research and development in this area.

The aim of this paper is to describe the European context related to healthcare, and its implications for cross-border healthcare in Europe. The background information demonstrates the need for further research and development in this area. As a response to this need, the European Commission funded the Methods of Assessing Response to Quality Improvement Strategies (MARQuIS) research project. This article comprises two main sections: a description of the European context, and a description of the MARQuIS project itself.

## THE EUROPEAN CONTEXT

Citizens are increasingly crossing borders within the European Union (EU). The vast majority travel for reasons unrelated to healthcare. In some cases, these movements give rise indirectly to demands on health services, and in others patients may directly seek specific care services in other member states.

Europeans have always been free to travel to receive care in other member states, but if they wished to benefit from their statutory social protection scheme, they were subject to their local or national legislation on social protection. According to article 22 of Regulation no. 1408/71, they could benefit from health services in another European Economic Area country, with access to emergency care during short-term stays. They needed, however, prior authorisation for elective care in another member state since the range of available benefits was limited to those covered in the country of insurance.^1^ This was challenged in 1991, when the European Court of Justice defined healthcare as a service.^2^ Consequently, in the well-known Kohll and Decker rulings of 1998, and subsequent cases, the European Court of Justice established a new type of cross-border access to health services in the EU. According to treaty principles of free movement of persons, goods and services, citizens should be able to go abroad for non-hospital care treatment at the expense of their statutory social protection scheme.^3^ Built on articles 28 and 30 (free movement of goods) and articles 49 and 50 (free movement of services) of the treaty, this jurisprudence urges member states to suppress barriers to fundamental freedoms. Those new rules apply to social insurance systems as well as to national health services, according to the Watts rulings of 16 May 2006.

The above jurisprudence started a debate on the right balance between different principles in European treaties: on the one hand, free movement of people, goods and services, and on the other hand the responsibility of member states to organise their healthcare systems. The healthcare field is essentially guided in the treaties by the rules of national sovereignty, onto which is grafted the community principle of subsidiarity:

“the Community only intervenes in those fields which are not part of its exclusive competence such as public health, if and to the extent that, because of their dimension or their effect, the objectives of the envisaged action would be better achieved at Community level.”

Except for particular instances, the level of suitable administration for a function remains the most decentralised level. The member states have thus decided that the state or regional level is the most appropriate for decisions regarding health. Consequently, Community actions with regard to social or health matters are only legitimate if they add to or strengthen those carried out at the national level. Based on this and up to now, the majority of Community legislation enacted in the field of health has not fundamentally transformed the way health systems operate in the countries of Europe, as the treaties have only given the EU very tenuous powers over health systems. This reflects the diversity that is at the very origin of European social systems, themselves reflected in the differences in the constitutional and administrative organisation of member states. And this diversity also holds within countries: the number of states in Europe which authorise regions to intervene appreciably in the field of health has markedly increased in the past 30 years. The content of regional health powers is itself variable. It may be minimal, as in the implementation of national health legislation and the management of part of the health system, or maximal, as in decision-making powers for regulation, health planning, financing and the supply of hospital care.

With particular reference to hospitals, major differences in systems may be measured with a few key elements. First, the means of access to hospitals can be very different from one member state to another. National hospital structures are also highly varied, especially in terms of the division between public and private, and within the private sector, between for-profit and non-for-profit organisations. As this concerns the burden of public hospital costs within the national total health budget, such differences in the organisation of systems cause significant budgetary differences. On further analysis, it is also clear that the models of medical organisation, internal management and decision-making powers in hospitals are very different from one member state to another.

Convergence is, however, growing for various reasons: cost containment, innovation, consumerism, ageing, etc. This is visible in policies for access, quality and financing of hospitals. The construction of the EU has itself been the basis of a number of actions, leading to a certain degree of convergence. Legislation is in place concerning drugs and medical devices, based on free movement of goods. The mutual recognition of professional qualifications is also particularly important in the healthcare sector, as a driving force of convergence. Article 152 of the Treaty of Amsterdam (1997) widened the powers of the Union with regard to public health by conferring on it genuine decision-making powers in certain fields. This is particularly true with regard to the quality and security of organs and substances of human origin and blood and its derivatives.

Regardless of increased reimbursement of care made possible under EU jurisprudence, cross-border cooperation has also developed between several member states—for example, Belgium and France, Germany and Luxembourg, and the Netherlands and the UK.^1^ With the financial help of INTERREG (Innovation & Environment Regions of Europe Sharing Solutions) programmes, initiatives to improve the social and economic situation were developed in border regions to facilitate the administrative procedures, and to extend contracts for providing benefits-in-kind across borders. Cross-border care also means mobility of health professionals, and the way this should be monitored to ensure quality of care.^4^ ^5^

In this context, patient mobility has become a relevant issue on the EU’s agenda. The need to respond to the jurisprudence of the European Court of Justice led to the creation of a high-level process of reflection on patient mobility in 2002 to discuss issues related to a growing number of patients in specific situations, including border regions, highly specialised care, tourists and people residing in another country. A high-level group on health services and medical care was set up in 2004 to follow developments. Various working groups prepared concrete proposals for the ministers of health about possible ways to improve cooperation and exchange of information between member states at the European level. In this high-level group, member states have been asked to debate issues of growing patient mobility, and to focus greater attention on cross-border healthcare. Some areas for attention thus far have been the provision of better information systems on spare capacity across the EU to reduce waiting lists for operations, conducting joint health technology assessment, creating European centres of reference for therapies involving advanced technologies and treatments for rare diseases, and the development of a common definition of rights, entitlements, and duties of patients at the European level.

- In June 2006, the ministers of EU member states approved the following values and principles for all member state health services:
- universality (access to healthcare must be ensured for every person living in the European Union);
- access to good quality care;
- equity (equal access to healthcare regardless of ethnicity, gender, age, social status and ability to pay)
- solidarity (linked to the financial schemes under which the health systems are funded).

The principles also state that reducing health inequalities must be one of the aims of health systems, as well as a shift towards preventive measures.

Following the exclusion of healthcare services from the Services Directive adopted in 2006, the European Commission was asked to work on a strategy for patient mobility that facilitated progress while respecting national responsibility for health systems. Again, the issue of the right balance between free movement and the responsibility of member states to protect their citizens and organise their healthcare systems was debated in the consultation process—which should lead to a directive in this field.

On a global level, trade in health services across borders have brought mixed benefits.^6^ The main concerns are that uncontrolled patient choice will damage some national health systems, treatments abroad will cost more and national authorities will be unable to regulate rates of treatment and spending, national health systems will be left with half-empty institutions, the system overall will become inefficient, medical staff will migrate across borders, and categories of care and entitlements will be defined differently internationally.^4^ Therefore, if trade in health services is to continue, policy makers must act to mitigate any adverse consequences and facilitate the gains.^4^

At the same time, healthcare systems in member states face increasing pressures and demands in terms of universal access to services, cost containment and sustainability of financing.^7^ This has led to the rapid growth of global interest in the evaluation of healthcare, due to the increasing need within individual countries to monitor the use of scarce resources to deliver healthcare, and the quality of those services.^8^ Increasing the value of health systems requires experimentation and performance measurement using actionable and specific indicators, benchmarking within and across borders, and the sharing of information, making further work at the international level imperative.^9^ Through international collaboration, experiences can be exchanged, providing evidence of what works and what does not, providing the knowledge for evidence-based practice.^10^

On the research side, the EU has funded a number of Scientific Support to Policies (SSP) activities within the Sixth Framework Programme, with the aim of providing the evidence needed by EU policy makers to deal with issues that European citizens face as a result of enhanced mobility in Europe. The Europe4Patients project (e4p, 2004–7) explored the impact of an integrated Europe on patients, in terms of the potential benefits through developments such as greater access to centres of excellence, and actions that overcome transfrontier imbalances between demand and supply. The Health Basket project (2004–7) intended to provide information to national and EU policy makers on reliable comparisons about available health services in European member states, how these are defined, what their costs are and the prices. The MARQuIS research project we present in this supplement is one of the SSP projects that focused on cross-border care.

## THE MARQUIS PROJECT

### Aim and conceptual framework

MARQuIS intends to assess the value of different quality strategies, and to provide the information needed when countries contract care for patients moving across borders, and when individual hospitals review the design of their quality strategies. The results are intended to provide evidence-based advice for the further development of formal quality procedures at the EU level either for healthcare institutions or for developing existing approaches.

The MARQuIS project has adopted a definition of cross-border care that includes five categories of mobile patients.^11^ Due to the difficulties in identifying the different categories in current healthcare databases, these five categories have been defined in descriptive terms for the project as follows:

- citizens who, while on holiday, need to use healthcare services in the country they are visiting. In these cases, there are arrangements throughout the European Economic Area (EEA) to facilitate the process, conferring the right to treatment during a temporary visit;
- citizens who retire to a different country and wish to use the healthcare system of the country where they are currently living;
- people sharing close cultural or linguistic links with the region where care is provided. This patient group also includes migrants returning to their country of origin to receive care;
- patients who cross a border to receive healthcare or to buy health goods. This is often because of perceived advantages related to quality, accessibility or prices, specifically out-of-pocket payments borne by patients;
- patients who are sent abroad by their own health system to overcome capacity restrictions at home. Some patients cross borders within the framework of cooperative agreements in order to share facilities, especially in relation to capital-intensive or highly specialised services.

**Figure 1:**
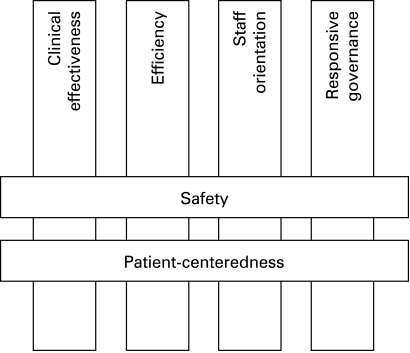
Dimensions of quality from the PATH theoretical model for hospital performance.^14^

To study quality of care in the MARQuIS project we chose the dimensions of quality of the PATH (Performance Assessment Tool for Quality Improvement in Hospitals) conceptual framework, developed by the World Health Organization (WHO) Regional Office for Europe,^12^ since it provides an up-to-date, comprehensive framework for hospitals, based on previous existing knowledge. The PATH conceptual framework advocates a multidimensional approach with six interrelated dimensions that should be assessed simultaneously. Two of these dimensions (safety and patient-centredness) cut across the other four dimensions (clinical effectiveness, efficiency, staff orientation and responsive governance), since they are inter-related. Figure 1 is a graphical representation of this framework. The MARQuIS project focuses mainly on the two central dimensions (safety and patient-centredness) due to their relevance and inter-relation with the other dimensions, and also explores clinical effectiveness.

### Methods

**Figure 2:**
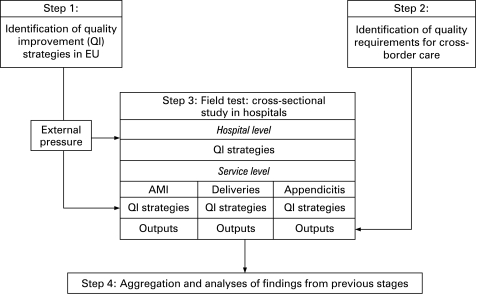
Scheme of the stages of MARQuIS project. AMI, acute myocardial infarction.

A multimethod approach involving qualitative and quantitative methods was chosen, and included literature review, qualitative studies, questionnaires and visits to centres to verify questionnaire data and obtain complementary information. To fulfil its objectives, the MARQuIS project was designed in four stages, to last for 3 years (2004–7, extended to June 2008). The information from each stage was to be used as the basis for the following stage. Figure 2 shows a basic scheme of the stages of the MARQuIS project.

#### First stage

The first stage of the project focused on the review and descriptive analysis of the current situation in Europe regarding, on the one hand, quality improvement and, on the other hand, cross-border care. To explore quality improvement, a survey was conducted of key experts in quality improvement from the 25 EU member states making up the EU in 2005 to gather information about views and accounts of quality improvement policies and strategies in different healthcare systems. To analyse cross-border care, directives of the EU with respect to patient safety, empowerment and access across borders were reviewed. Data on foreign admissions were collected to identify quantity and type of cross-border care occurrence in Europe. Additional information about this stage is described by Spencer and Walshe.^13^

#### Second stage

**Key messages**

- Citizens are increasingly crossing borders within the EU
- In the EU, health planning, organisation and delivery are the responsibility of member states. Intervention by the EU is based on the principle of subsidiarity, and covers areas such as public health or legislation on general interest topics such as drug approval
- In 1991 the European Court of Justice defined healthcare as a service, and started a debate on the balance between the free movement of persons, goods and services (established in EU treaties) versus the responsibility of member states to organise their healthcare systems
- The EU funded a number of Scientific Support to Policies (SSP) research projects within the Sixth Framework Programme, with the aim of providing the evidence needed by EU policy makers to deal with decisions faced as a result of enhanced mobility in Europe
- MARQuIS is one such project, which aimed to assess the value of different quality strategies, and to provide the information needed when countries contract care for patients moving across borders, and when individual hospitals review the design of their quality strategies
- The project involved 389 hospitals in eight countries and included an audit via on-site visits to 89 of them

The second stage focused on the identification of key requirements for securing patient empowerment and safety in hospital care. To this end we reviewed some EU directives pertaining to patient safety, empowerment and access across borders. Furthermore, a qualitative study with semi-structured interviews of patients, professionals and financers was carried out to explore their views regarding quality requisites—mainly related to safety and patient-centredness—when receiving or providing cross-border care.^14^ Finally, an exploratory study was done to estimate volume and determine the most common conditions that led to hospitalisation of European cross-border patients.^15^

#### Third stage

The third stage of the project built on the information collected in the two previous stages of the project, and aimed to describe in a sample of states how hospitals have applied national quality strategies, how far they meet the defined requirements of cross-border patients, and what variables of organisation and methodology are associated with meeting these requirements. The information was collected during the MARQuIS field test, which consisted of two main phases. In the first phase a cross-sectional survey of hospitals was done with a self-administered questionnaire. In the second phase an on-site audit was done of a sample of the hospitals that participated in the questionnaire survey. Both the questionnaire and the audit examined quality improvement at two levels: hospital management and ward. Three wards were selected based on the most frequent diagnoses identified in cross-border care in previous stages of the project: acute myocardial infarction, deliveries and appendicitis. Eight European countries participated in the field test: Belgium, Czech Republic, France, Ireland, Poland, Spain, the Netherlands, and the UK. These countries were selected mainly based on feasibility characteristics: a national agency with healthcare assessment experience and a solid reputation in the country was needed to be able to promote hospital participation in the study, and to perform assessments of hospitals. The methods for the field test are described elsewhere.^16^ ^17^

#### Fourth stage

The fourth stage consisted of the aggregation and analyses of findings from all previous stages in order to draw conclusions and develop recommendations to policy makers at the EU as well as the national level. Recommendations for healthcare organisations and professional organisations were also drawn from the information and distributed for consultation to member states and key stakeholders.^18^

### Results

The MARQuIS project collected self-reported data from 389 hospitals in eight European countries, and included an external assessment of 89 of these hospitals. In all, the study involved 16 European organisations (universities, accreditation bodies, scientific societies and federations) related to healthcare quality, and more than 30 professionals. In addition to the professionals directly involved in the project, an advisory council of more than 15 international experts provided recommendations to the research team. The scientific results and recommendations of this project are presented here.

## CONCLUSION

The successful completion of this project can be considered an accomplishment in itself. The multinational and multicultural approach favoured international information exchange, which in itself can contribute to self-learning and homogenisation of the work in different countries. This self-learning and homogenisation process seems crucial in the evolving European context. For this reason the authors advocate the promotion of further international research focused on the quality of European healthcare.
